# A bicoherence approach to analyze multi-dimensional cross-frequency coupling in EEG/MEG data

**DOI:** 10.1038/s41598-024-57014-0

**Published:** 2024-04-11

**Authors:** Alessio Basti, Guido Nolte, Roberto Guidotti, Risto J. Ilmoniemi, Gian Luca Romani, Vittorio Pizzella, Laura Marzetti

**Affiliations:** 1 Department of Neuroscience, Imaging and Clinical Sciences, “G. d’Annunzio” University of Chieti-Pescara, 66100 Chieti, Italy; 2 Department of Neurophysiology and Pathophysiology, University Medical Center Hamburg-Eppendorf, 20246 Hamburg, Germany; 3 Department of Neuroscience and Biomedical Engineering, Aalto University School of Science, 02150 Espoo, Finland; 4 BioMag Laboratory, HUS Medical Imaging Center, University of Helsinki, Aalto University and Helsinki University Hospital, 00029 Helsinki, Finland; 5 Institute for Advanced Biomedical Technologies, “G. d’Annunzio” University of Chieti-Pescara, 66100 Chieti, Italy

**Keywords:** Computational neuroscience, Statistics

## Abstract

We introduce a blockwise generalisation of the Antisymmetric Cross-Bicoherence (ACB), a statistical method based on bispectral analysis. The Multi-dimensional ACB (MACB) is an approach that aims at detecting quadratic lagged phase-interactions between vector time series in the frequency domain. Such a coupling can be empirically observed in functional neuroimaging data, e.g., in electro/magnetoencephalographic signals. MACB is invariant under orthogonal trasformations of the data, which makes it independent, e.g., on the choice of the physical coordinate system in the neuro-electromagnetic inverse procedure. In extensive synthetic experiments, we prove that MACB performance is significantly better than that obtained by ACB. Specifically, the shorter the data length, or the higher the dimension of the single data space, the larger the difference between the two methods.

## Introduction

The development of multivariate (MV) data-analysis tools is crucial in most scientific fields involving data collection and interpretation of complex systems. Although each particular field benefits from specific MV methods, a fundamental role is played by techniques that aim at analysing statistical dependencies between subsystems, i.e., groups of variables. These approaches allow one to detect regularities that may help suggest theoretical explanations of the underlying phenomena, thus potentially filling knowledge gaps and enabling one to answer novel questions without the need to rely on potentially suboptimal dimensionality reductions^1^.

One such complex system is undoubtedly the human brain^2^. Thus, a field with a currently growing interest in exploiting these MV methods (also termed in literature as multi-dimensional, MD, or blockwise^3,4^) is functional neuroimaging, the discipline that focuses on explaining brain functioning by means of the analyses of measured neuronal data. The human brain is an integrated physical system that gives rise to cognitive functions and complex behavior by relying (together with other fundamental physiological mechanisms) on communication among groups of neurons^5,6^. In particular, the synchronization of electrical activity within and between neuronal assemblies has been postulated to be essential for effectively allowing this communication^7^. In this context, being able to robustly detect the presence of statistical dependencies between time series associated with different brain regions allows one to provide important insight into its functioning; merely analyzing the activity of the regions separately fails to provide the information conveyed by such interdependencies^8^. Specifically, the subfield of functional neuroimaging interested in analysing those couplings is the study of functional connectivity^9^.

Among the noninvasive neuroimaging techniques that capture electromagnetic neuronal oscillations (generated by microscopic neuronal activity) for computing functional connectivity, electroencephalography (EEG) and magnetoencephalography (MEG) are considered the state of the art^10,11^. Several studies have demonstrated how the macroscopic EEG/MEG oscillatory patterns can (empirically) show functional dependencies at the same or at different frequencies, i.e., cross-frequency relationships^12–14^ (for other references, see Palva et al.^15^). It has been hypothesised that different coupling modalities (e.g., same vs. cross-frequency interactions, and phase vs. amplitude couplings) may denote different mechanisms used for integrating information across distinct spatial and temporal scales, and thus subserve separate cognitive functions^16,17^.

In the last 2 decades, dozens of EEG/MEG functional connectivity methods have been defined^11^, but the majority of them are one-dimensional (1D), i.e., they can only characterize dependencies between two (scalar) univariate time series at the time. However, simulation and real-data studies have pointed out that applying 1D methods may be suboptimal for the analysis of functional connectivity (e.g.,^3^), thus leading to increased efforts in generalizing the standard methods to the MD case (e.g.,^18–21^). For instance, Pascual-Marqui^18^ introduced a frequency-domain method for investigating linear MD dependencies based on the second-order statistical moments. Similarly, Ewald et al.^19^ introduced methods (sharing similarities with canonical correlation analyses) able to catch linear frequency-specific phase coupling from the cross-spectral matrix of the data. In Basti et al.^20^, by linear transforming the original multivariate time series through a frequency-domain spatial whitening and by applying an averaging process, the authors derived an MD estimator of directionality of frequency-specific dependencies based on the phase slopes of cross-spectral quantities. Bruna and Pereda^21^ generalized an index termed as phase-locking value (and based on the mean resultant length of the instantaneous phase difference between time series filtered at the same frequency^22^) through an eigendecomposition approach. The majority of MD methods are able to catch only dependencies between oscillations at the same frequency. One notable exception can be found in Soto et al.^23^, where the authors exploited a canonical correlation analysis to identify cross-frequency correlations in MEG data and pinpoint the frequencies responsible for such correlations. Nevertheless, this method has been formulated to detect only dependencies between neural power at different frequencies. That is, the presence of MD phase couplings cannot be investigated by such a method. Furthermore, cross-frequency interactions might result in a symmetric cross-covariance, leading to a theoretical difficulty in being detected by a second-order statistical method.

The aim of this paper is to introduce an MD version of the antisymmetric cross-bicoherence^24^, a 1D approach based on third-order statistical (bispectral) analysis and defined to detect nonlinear (quadratic) phase coupling in the frequency domain. Due to its MD nature, this method allows avoiding the application of dimensionality reductions, preventing the loss of information that makes 1D methods suboptimal. In the “Methods” section, we will also prove some fundamental properties of this method, such as its invariance under orthogonal transformations of the data. Finally, by relying on three different synthetic experiments, we will compare our novel approach with the 1D version methodology.

## Methods

### Theoretical formulation

Let $f_1$, $f_2 \in {\mathbb {R}}$ and *x*, *y*, $z\in {\mathbb {R}}^T$, where $T\in {\mathbb {N}}$, denote two frequencies and three scalar time series of length *T*. ${\hat{\cdot }}$ and $\left\langle {\cdot }\right\rangle$ will denote, respectively, the Fourier transform and the mean across segments to which the data are divided (or the expected value across data segments for time series of infinite length). The cross-bispectrum, i.e., the frequency-domain counterpart of the third-order moment, for the above defined time series and frequencies is

1 $$\begin{aligned} B_{x,y,z}(f_1,f_2):=\left\langle {{\hat{x}}(f_1) {\hat{y}}(f_2) {\hat{z}}(f_1+f_2)^* }\right\rangle , \end{aligned}$$

where $*$ indicates complex conjugate. Bispectral analysis can reveal the presence of a nonlinear (quadratic) interaction between time series. A typical analysis aims at investigating the dependency between only two time series by using, in the computation of bispectrum, as *y* the first time series *x*, i.e., $B_{x,x,z}(f_1,f_2)$. In order to better understand the type of statistical dependency caught by such an analysis, let us consider only two scalar time series, *x* and *z*, where $x(t)=a\textrm{cos}(2\pi f t )$ with $a\in {\mathbb {R}}$, and *z* is a squared copy of *x*. The bispectrum at the frequencies $f_1=f_2:=\nu$, $B_{x,x,z}(\nu ,\nu )$, is then $\left\langle {{\hat{x}}(\nu )^2{\hat{z}}(2\nu )^* }\right\rangle$ = $k\left\langle {\delta (\nu -2\pi f)^2\delta (2\nu -4\pi f) }\right\rangle$, with *k* being a constant; thus, $B_{x,x,z}(\nu ,\nu )$ differs from 0 if and only if $\nu =f$. On the contrary, a linear coupling analysis applied to the two time series would not detect any dependency.

From here on, we will omit explicitly writing the frequency dependence of the above quantities for the ease of reading.

As shown in Eq. (1), and similarly for the covariance matrix and for the cross-spectrum (i.e., the frequency domain counterpart of the second-order moment), the cross-bispectrum is a quantity whose magnitude depends on the amplitudes of the measured signals. In order not to be strongly driven by the amplitude of the time series, a relative quantity $b_{x,y,z}:=|B_{x,y,z}/N_{x,y,z}|$ (termed as cross-bicoherence), for a suitable term $N_{x,y,z}$, is usually used to assess the strength of coupling between the phases of the time series *x* and *y* at two possibly different frequencies with respect to the phase of the time series *z* at a third frequency that is equal to the sum of the other two. This normalisation step resembles the procedure that defines the correlation/coherence as normalised versions of the covariance/cross-spectrum, leading to methods that are only weakly dependent on the amplitudes and thus suitable to assess the (phase) dependency of interest. As opposed to the normalisation of the latter (linear) methods, the approach for the cross-bispectrum is non-trivial and not completely satisfactory^25^. Indeed, different choices can be found in literature, each of which shows different pros and cons. For instance, the standard normalization term $N_{x,y,z}$^26^ corresponds to the product among the square root of the power spectrum of the three time series at the frequencies used in Eq. (1). A significant drawback of this choice is that it would complicate the interpretation since the bicoherence would not be bounded. Another common choice is that of dividing the cross-bispectrum by $N_{x,y,z}=(\left\langle {|{\hat{x}} {\hat{y}}|^2 }\right\rangle )^{1/2} (\left\langle {|{\hat{z}}|^2 }\right\rangle )^{1/2}$^27^. This approach is known as bivariate normalisation. It is important to notice that, thanks to the Cauchy–Schwarz Theorem, $b_{x,y,z}\le 1$ in this case. However, the term may depend on the coupling between *x* and *y*. Other choices are the trivariate normalisation^28^ and the univariate one^25^. In particular, the univariate approach avoids the dependency on the coupling between two time series. The normalization that better fits our purpose of defining a multidimensional quantity that is invariant under orthogonal transformations of the data is the bivariate normalization, which will thus be employed in the following.

As defined above, cross-bicoherence can be exploited to assess the presence of non-linear relationships between time series. For instance, in the neuroimaging field, cross-bicoherence can detect significant functional coupling between neuronal (e.g., electro/magnetoencephalography or EEG/MEG-acquired) data^29,30^ associated either with two sensors or with two regions/locations in the cortex after the application of a method to solve the electromagnetic inverse problem^31^. Nevertheless, cross-bicoherence is not robust against instantaneously correlated noise that may affect the data and induce an artefactual apparent coupling, thus inducing false positive results that suggest the presence of an underlying statistical dependency when this is not the case. In neuroimaging, this issue arises due to the volume conduction effects, which cannot be completely removed through solving the bio-eletromagnetic inverse problem. For this reason, a modified version of the cross-bicoherence has been introduced^24,32^. This approach is termed as antisymmetric cross-bicoherence (ACB) and is defined as

2 $$\begin{aligned} ACB_{x,y,z}:=\frac{\left| B_{x,y,z}-B_{z,y,x}\right| }{N_{x,y,z}+N_{z,y,x}}, \end{aligned}$$

namely, the normalised difference between two cross-bispectra with the application of an index permutation. A non-null ACB, Eq. (2), cannot result from a superposition of independent signal sources and, therefore, it cannot reflect an instantaneously-correlated artifactual statistical dependency between independent sources (e.g., brain areas). We chose to take advantage of this property of ACB, and to generalise its definition in order to introduce a quantity that can take as input two multivariate time series. The generalization will be such that certain fundamental properties will hold: (1) for a particular linear mixture of independent sources and an infinite amount of data, it is equal to 0; (2) it is upper-bounded by 1; (3) it is invariant under orthogonal transformations of the two multivariate time series; (4) it has an equivalent formulation in terms of the Kronecker product between frequency-domain-transformed data and their cross-spectra. For this purpose, instead of three scalar signals, let us now consider three vector time series $X=(x_1, \ldots ,x_{N_x})'\in {\mathbb {R}}^{N_x\times T}$, $Y=(y_1, \ldots ,y_{N_y})'\in {\mathbb {R}}^{N_y\times T}$ and $Z=(z_1, \ldots ,z_{N_z})'\in {\mathbb {R}}^{N_z\times T}$, where $N_x$, $N_y$ and $N_z$ are natural numbers, and introduce the multi-dimensional antisymmetric cross-bicoherence (MACB) as

3 $$\begin{aligned} \text {MACB}_{X,Y,Z}:=\sqrt{\frac{\sum _{i,j,k} \left| B_{x_i,y_j,z_k}-B_{z_k,y_j,x_i}\right| ^2}{2\sum _{i,j,k}\left( N_{x_i,y_j,z_k}^2+N_{z_k,y_j,x_i}^2\right) }}. \end{aligned}$$

The electrophysiological sensor level data, such as the measured EEG or MEG signals, can be considered as a linear mixture of brain electrical signals. Let us heuristically see that a non-vanishing MACB cannot be generated from independent sources. If each spatial component of *X*, *Y* and *Z* is a combination of the realizations (considered as having an infinite length) of *M* independent neural sources, each term within the finite sum in the MACB numerator (Eq. (3)) $B_{x_i,y_j,z_k}-B_{z_k,y_j,x_i}0$ is equal to 0, indeed, $B_{x_i,y_j,z_k}(f_1,f_2)=\sum _{m}c^x_{i,m}c^y_{j,m}c^z_{k,m}\left\langle {{\hat{s}}_m(f_1) {\hat{s}}_m(f_2) {\hat{s}}_m(f_1+f_2)^* }\right\rangle + \textrm{coupling}$ $\textrm{terms}$, but the “coupling terms” are null because of the hypotheses applied to all the $\left\langle {{\hat{s}}_p(f_1) {\hat{s}}_q(f_2) {\hat{s}}_r(f_1+f_2)^* }\right\rangle$ terms, where at least one of the three subscripts *p*, *q* and *r* is different from the other two^32^. The remaining part is symmetric with respect to permutation of the subscripts and cancels out in the difference between $B_{x_i,y_j,z_k}$ and $B_{z_k,y_j,x_i}$. $\text {MACB}_{X,Y,Z}$ is thus equal to 0 since it corresponds to the square root of the sum of the magnitude of complex numbers equal to 0. Let us now prove three propositions regarding some properties of MACB:

#### Proposition 1

$\text {MACB}_{X,Y,Z}$ is upper-bounded by 1.

#### Proof

$$\begin{aligned} \text {macb}_{X,Y,Z}^2= & {} \frac{\sum _{i,j,k} \left| B_{x_i,y_j,z_k}-B_{z_k,y_j,x_i}\right| ^2}{2\sum _{i,j,k} \left( N_{x_i,y_j,z_k}^2+N_{z_k,y_j,x_i}^2\right) }\\= & {} \frac{\sum _{i,j,k} \left( |B_{x_i,y_j,z_k}|^2+|B_{z_k,y_j,x_i} |^2-B_{x_i,y_j,z_k}^*B_{z_k,y_j,x_i}-B_{x_i,y_j,z_k}B_{z_k,y_j,x_i} ^*\right) }{2\sum _{i,j,k} \left( N_{x_i,y_j,z_k}^2 +N_{z_k,y_j,x_i}^2\right) }\\\le & {} \frac{ 2\sum _{i,j,k}\left( |B_{x_i,y_j,z_k}|^2 +|B_{z_k,y_j,x_i}|^2\right) }{2\sum _{i,j,k} \left( N_{x_i,y_j,z_k}^2+N_{z_k,y_j,x_i}^2\right) } \le \frac{\sum _{i,j,k} \left( N_{x_i,y_j,z_k}^2 +N_{z_k,y_j,x_i}^2\right) }{\sum _{i,j,k} \left( N_{x_i,y_j,z_k}^2+N_{z_k,y_j,x_i}^2\right) }=1 \end{aligned}$$

To obtain the above result, we used the following identity and inequalities:

- $$|a-b|^2=|a|^2+|b|^2-a'b-ab'$$, $$a, b\in {\mathbb {C}}$$;
- $$|a|^2+|b|^2 \ge \pm (a'b+ab')$$, $$a, b\in {\mathbb {C}}$$;
- Cauchy–Schwarz inequality ($A'B\le ||A||||B||$, with *A*, *B* vectors of suitable dimension). □

The fact that the value of MACB is always lower than 1 guarantees that it does not indefinitely increase with the increase of the amplitudes in the time series.

#### Proposition 2

$\text {MACB}_{X,Y,Z}=\text {MACB}_{UX,VY,WZ}$ with $U\in {\mathbb {R}}^{N_x\times N_x}$, $V\in {\mathbb {R}}^{N_y\times N_y}$, and $W\in {\mathbb {R}}^{N_z\times N_z}$ being three orthogonal linear matrix transformations.

#### Proof

Let us separately prove that both the numerator and the denominator are invariant under orthogonal transformation of the data.

Numerator:

Each of the four terms included within the sum across the subscripts *i*, *j* and *k*, i.e., $|B_{x_i,y_j,z_k}|^2$, $|B_{z_k,y_j,x_i}|^2$, $B_{x_i,y_j,z_k}^*B_{z_k,y_j,x_i}$ and $B_{x_i,y_j,z_k}B_{z_k,y_j,x_i}^*$ (see Proposition 2 to see why those four terms appear), has the following form: $P_{i,j,k}Q_{i,j,k}$ where *P* and *Q* are third-order real tensors.

It is thus sufficient to show that the quantity defined above is invariant under orthogonal transformations (let us call the transformation matrices *U*, *V* and *W* and the tensors as $\tilde{P}$ and ${\tilde{Q}}$).

$$\begin{aligned} \sum _{i,j,k}{\tilde{P}}_{i,j,k}{\tilde{Q}}_{i,j,k}= & {} \sum _{i,j,k} \sum _{m_1,m_2,m_3}\sum _{n_1,n_2,n_3} U_{i,m_1}V_{j,m_2}W_{k,m_3} P_{m_1,m_2,m_3}U_{i,n_1}V_{j,n_2}W_{k,n_3}Q_{n_1,n_2,n_3}\\= & {} \sum _{m_1,m_2,m_3}\sum _{n_1,n_2,n_3} \sum _{i}U_{i,m_1} U_{i,n_1}\sum _{j}V_{j,m_2}V_{j,n_2}\sum _{k}W_{k,m_3}W_{k,n_3} P_{m_1,m_2,m_3}Q_{n_1,n_2,n_3}\\= & {} \sum _{m_1,m_2,m_3}\sum _{n_1,n_2,n_3} \delta _{m1,n1} \delta _{m2,n2}\delta _{m3,n3}P_{m_1,m_2,m_3}Q_{n_1,n_2,n_3}. \end{aligned}$$

By simplifying the terms included in the sums using the properties of the Kronecker $\delta$ term, and by changing notation, the previous quantity becomes

$$\begin{aligned} \sum _{m_1,m_2,m_3}P_{m_1,m_2,m_3}Q_{m_1,m_2,m_3} =\sum _{i,j,k}P_{i,j,k}Q_{i,j,k}. \end{aligned}$$

Denominator:

Contrary to the terms included in the numerator, those included in the denominator of MACB are slightly different, so we prefer to treat them differently. Let us consider only first term (the second term behaves similarly) and use the transformed vectors *UX*, *VY*, *WZ* to see the independence on *U*, *V*, and *W*.

$$\begin{aligned} \sum _{i,j,k} N_{U_i' X,V_j' Y,W_k' Z}^2= & {} \sum _{i,j,k} (\left\langle {U_i' {\hat{X}} V_j' {\hat{Y}}(U_i' {\hat{X}} V_j' {\hat{Y}})^* }\right\rangle )(\left\langle {W_k' {\hat{Z}}(W_k' {\hat{Z}})^* }\right\rangle )\\= & {} \sum _{i,j,k} (\left\langle {U_i' {\hat{X}} V_j' {\hat{Y}}(U_i' {\hat{X}} V_j' {\hat{Y}})^* }\right\rangle )(W_k'\left\langle { {\hat{Z}}{\hat{Z}}^H }\right\rangle W_k)\\= & {} \sum _{i,j} (\left\langle {U_i' {\hat{X}} V_j' {\hat{Y}}(U_i' {\hat{X}} V_j' {\hat{Y}})^* }\right\rangle )tr(\left\langle { {\hat{Z}}{\hat{Z}}^H }\right\rangle )\\= & {} \sum _{i,j} \left\langle {U_i' {\hat{X}} V_j' {\hat{Y}} {\hat{Y}}^H V_j {\hat{X}}^H U_i }\right\rangle tr(\left\langle { {\hat{Z}}{\hat{Z}}^H }\right\rangle )=\sum _{i} \left\langle {U_i' {\hat{X}} tr\left( {\hat{Y}} {\hat{Y}}^H\right) {\hat{X}}^H U_i}\right\rangle tr(\left\langle { {\hat{Z}}{\hat{Z}}^H }\right\rangle )\\= & {} \left\langle {\left( \sum _{i}U_i' {\hat{X}} {\hat{X}}^H U_i\right) tr\left( {\hat{Y}} {\hat{Y}}^H\right) }\right\rangle tr(\left\langle { {\hat{Z}}{\hat{Z}}^H }\right\rangle )= \left\langle {tr\left( {\hat{X}} {\hat{X}}^H \right) tr\left( {\hat{Y}} {\hat{Y}}^H\right) }\right\rangle tr(\left\langle { {\hat{Z}}{\hat{Z}}^H }\right\rangle ) \end{aligned}$$

which is independent on *U*, *V* and *W*. The same holds for the second term of the sum, and thus the denominator, and the whole MACB, is invariant with respect to orthogonal transformation of the data. Finally, by using the linearity of the trace and that $tr(M_1)tr(M_2)=tr(M_1\otimes _{kr}M_2)$, where $\otimes _{kr}$ is the Kronecker product,

$$\begin{aligned} \left\langle {tr\left( {\hat{X}} {\hat{X}}^H \right) tr\left( {\hat{Y}} {\hat{Y}}^H\right) }\right\rangle tr(\left\langle { {\hat{Z}}{\hat{Z}}^H }\right\rangle )=tr\left( \left\langle {{\hat{X}} {\hat{X}}^H \otimes _{kr}{\hat{Y}} {\hat{Y}}^H }\right\rangle \otimes _{kr} \left\langle { {\hat{Z}}{\hat{Z}}^H }\right\rangle \right) . \end{aligned}$$

This property makes, e.g., MACB invariant under rotations of the data spaces. Such an invariance plays an important role in neuroimaging when estimating functional connectivity at the level of the cortex, e.g., in EEG or MEG. Indeed, in order to infer the three-dimensional neuronal activities by solving an electromagnetic inverse problem^10^, a coordinate system in the source space has to be defined; nevertheless, the choice of this system is arbitrary, and thus a method that is independent of this choice is fundamental. Let us make an example and consider the time series associated with two sources, one perfectly aligned with the z-axis and the other with the x-axis. Let us assume that in the canonical xyz-coordinate system $\{(1,0,0),(0,1,0),(0,0,1)\}$ the values of the time series are (1, 0, 1) and (0, 1, 1), respectively. Now, by applying an orthogonal transformation to the coordinate system that only inverts the orientation of the z-axis, we have that the values of the first time series change from (1, 0, 1) to $(-1,0,-1)$. While the covariance between the two time series in the first coordinate system is $-0.17$, in the transformed system, it becomes 0.17. This highlights that the covariance is not invariant to orthogonal changes in the data. Notably, it’s not solely a matter of sign, as the values can vary significantly based on the transformation, which emphasize the importance of defining and using a method that is invariant, such as MACB. If MACB were not invariant, it would have different values simply by considering, in the inverse process, the positive direction of the z-axis as the one going from top to bottom instead of the one going from bottom to top. $\square$

#### Proposition 3

$\text {MACB}_{X,Y,Z}$ can also be written as

$$\begin{aligned} \text {MACB}_{X,Y,Z}=\frac{||\left\langle {{\hat{X}} {\hat{Y}}' \otimes _{kr} {\hat{Z}}^H }\right\rangle -\left( \left\langle {{\hat{Y}} {\hat{X}}^H \otimes _{kr}{\hat{Z}} }\right\rangle \right) '||_F}{\sqrt{2tr\left( \left\langle {C_X \otimes _{kr}C_Y }\right\rangle \otimes _{kr}\left\langle {C_Z }\right\rangle \right) +2tr\left( \left\langle {C_Z \otimes _{kr}C_Y }\right\rangle \otimes _{kr}\left\langle {C_X }\right\rangle \right) }} \end{aligned}$$

where $C_{*}$ and *tr* denote the single-segment cross-spectral matrices and the trace.

#### Proof

Since we already proved the validity of the final form of the denominator, we can focus on the numerator. Let us take the six complex vectors used in the step 3 of the proof, i.e., *A*, $D \in {\mathbb {C}}^{N_x \times 1}$, *B*, $E \in {\mathbb {C}}^{N_y \times 1}$ and *C*, $G \in {\mathbb {C}}^{N_z \times 1}$. The (*i*, *j*) element of the matrix $M=AB'$ is equal to $a_ib_j$ and the $(i,N_z(j-1)+k)$ element of $S=M\otimes _{kr}C^H$ is equal to $M_{i,j}c_k^*=a_ib_jc_k^*$. Instead, the (*j*, *i*) element of $M=ED^H$ is equal to $e_jd_i^{*}$ and the $(N_z(j-1)+k,i)$ element of $S=M\otimes _{kr}G$ is $M_{j,i}g_k=e_jd_i^{*}g_k=g_ke_jd_i^{*}$. Now it is sufficient to recall the definition of the Frobenius norm of a matrix *S*, i.e., $\left\| {S} \right\| _F=\left( \sum _{i,j} |S_{i,j}|^2\right) ^{1/2}$, and apply this norm to the difference between the first *S* matrix and the transpose of the second *S* matrix (computed with $A={\hat{X}}(f_1)$, $B=E={\hat{Y}}(f_2)$, $C={\hat{Z}}(f_1+f_2)$, $D={\hat{X}}(f_1+f_2)$ and $G={\hat{Z}}(f_1)$). $\square$

### Expected value approximation in a Gaussian case

Our aim now is to define a reasonable higher bound for the expected value of MACB in case of independent zero-mean Gaussian data with *K* segments/trials each. For simplicity, let us consider the squared MACB among three scalar signals *x*, *y* and *z*, the MD generalisation will follow easily. The squared MACB is basically a ratio, and using the first-order bivariate Taylor expansion, we can approximate the expected value of a ratio, i.e., ${\mathbb {E}}(A/B)$, with the ratio between two expected values, i.e., ${\mathbb {E}}(A)/{\mathbb {E}}(B)$. Note: in order not to cause confusion between the expected value across signal realizations and the mean across (finite or infinite) trials/segments, we use here the symbol ${\mathbb {E}}$ to indicate the first one.

The expected value of the denominator (${\mathbb {E}}(B)$ in the example above) is ${\mathbb {E}}\left( 2\left( N_{x,y,z}^2+N_{z,y,x}^2\right) \right) =2{\mathbb {E}}\left( N_{x,y,z}^2\right) +2{\mathbb {E}}\left( N_{z,y,x}^2\right)$. For the first term (the second one is similar),

$$\begin{aligned} 2\frac{1}{K}\sum _{k}{\mathbb {E}}(|x(k,f_1)|^2){\mathbb {E}} (|y(k,f_2)|^2)\frac{1}{K}\sum _{l}{\mathbb {E}}(|z(k,f_1+f_2)|^2) =2\sigma _{x,f_1}^2\sigma _{y,f_2}^2\sigma _{z,f_1+f_2}^2. \end{aligned}$$

The expected value of the numerator (${\mathbb {E}}(A)$ above) is composed of four terms:

$$\begin{aligned} {\mathbb {E}}\left( \left| B_{x,y,z}-B_{z,y,x}\right| ^2\right)= & {} {\mathbb {E}}(|B_{x,y,z}|^2+|B_{z,y,x}|^2-B_{x,y,z}B_{z,y,x} ^{*}-B_{x,y,z}^{*}B_{z,y,x})\\= & {} {\mathbb {E}}(|B_{x,y,z}|^2)-{\mathbb {E}}(B_{x,y,z}B_{z,y,x}^{*}) -{\mathbb {E}}(B_{x,y,z}^{*}B_{z,y,x})+{\mathbb {E}}(|B_{z,y,x}|^2) \end{aligned}$$

For the first one (the fourth term is similar),

$$\begin{aligned} {\mathbb {E}}(|B_{x,y,z}|^2)= & {} \frac{1}{K^2}\sum _{k,l} {\mathbb {E}}(x(k)y(k)z(k)^{*}x(l)^{*}y(l)^{*}z(l)) =\frac{1}{K^2}\sum _{k}{\mathbb {E}}(|x(k)|^2) {\mathbb {E}}(|y(k)|^2){\mathbb {E}}(|z(k)|^2)\\= & {} \frac{1}{K}\sigma _{x,f_1}^2\sigma _{y,f_2}^2 \sigma _{z,f_1+f_2}^2. \end{aligned}$$

Second term (the third one behaves similarly): here, in order to see the independency among the terms, it is better to explicitate the dependency on the frequencies:

$$\begin{aligned} {\mathbb {E}}(B_{x,y,z}B_{z,y,x}^{*}) =\frac{1}{K^2}\sum _{k,l}{\mathbb {E}}(x(k,f_1)y(k,f_2) z(k,f_1+f_2)^{*}z(l,f_1)y(l,f_2)x(l,f_1+f_1)^{*})=0. \end{aligned}$$

Therefore, the expected value of the squared MACB (by using the linearity of the mean) among *X*, *Y* and *Z* can be approximated as:

$$\begin{aligned} {\mathbb {E}}(\text {MACB}_{X,Y,Z}^2)\approx \frac{\sum _{i,j,k}\left( \sigma _{x_i,f_1}^2 \sigma _{y_j,f_2}^2\sigma _{z_k,f_1+f_2}^2 +\sigma _{z_k,f_1}^2\sigma _{y_j,f_2}^2 \sigma _{x_i,f_1+f_2}^2\right) }{2K\sum _{i,j,k} \left( \sigma _{x_i,f_1}^2\sigma _{y_j,f_2}^2 \sigma _{z_k,f_1+f_2}^2+\sigma _{z_k,f_1}^2 \sigma _{y_j,f_2}^2\sigma _{x_i,f_1+f_2}^2\right) } =\frac{1}{2k}. \end{aligned}$$

Finally, by using the Jensen’s inequality for concave functions (applied to the approximation of the expected values), we have: ${\mathbb {E}}(\text {MACB}_{X,Y,Z})\le \sqrt{{\mathbb {E}}(\text {MACB}_{X,Y,Z}^2)}=1/\sqrt{2K}$, which denotes the sought bound.

**Figure 1:**
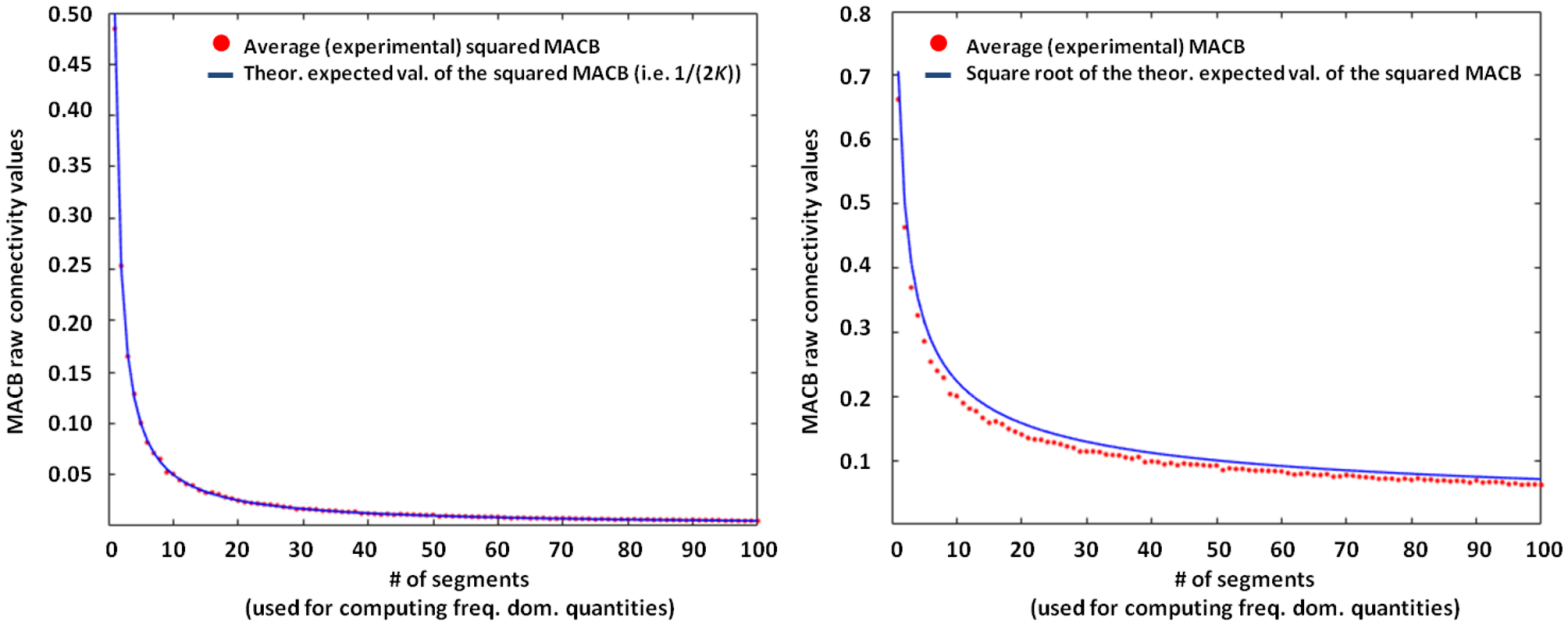
Difference between the experimental (red dotted line) and the theoretical (blue line) approximated bias for the squared MACB (left panel) and for the MACB (right panel). The right panel shows the value of $1/\sqrt{2K}$, where *K* is the number of segments in which the data are divided, is a reasonable higher bound for the actual bias.

Let us now computationally prove the validity of the bound defined above. Let (for each segment) ${\hat{X}}(f_1)$, ${\hat{Y}}(f_2)$, ${\hat{Z}}(f_1+f_2)$, ${\hat{X}}(f_1+f_2)$ and ${\hat{Z}}(f_1)$) be five vectors whose elements are taken from the standard complex Gaussian distribution. For 1000 different repetitions and a dimension of the three subspaces equal to 1 (i.e., *X*, *Y* and *Z* are three scalar time series), it is computationally evident (left panel of the Fig. 1) that the bias of the squared MACB linearly decreases as described by the estimation. The fact that $1/\sqrt{2K}$ is a (reasonable) higher bound is also evident from the simulations shown in the right panel of the Fig. 1.

## Materials

To understand whether MACB allows one to obtain better performance than those obtained by its one-dimensional version (i.e., ACB) in detecting the presence of cross-frequency pairwise (i.e., region *X* vs. region *Z*) couplings, we performed three simulation studies. We compared the performance of $\textrm{MACB}_{X,X,Z}$ and $\textrm{ACB}_{X,X,Z}$. Synthetic data were generated through MATLAB code; no real (animal/human) data have been used in the study.

The first synthetic experiment focused on the difference between the two methods as a function of the level of noise in a model mimicking a realistic neuroscientific scenario. This model also takes into account solutions of the forward and (electromagnetic) inverse problems. This experiment also aims at demonstrating the robustness of MACB to detect truly interacting sources in the presence of other active but non-interacting sources.

In the second experiment, we analysed the differences as functions of the dimensionality of the data spaces; from a neuroscientific perspective, this case simulates the presence of brain regions (also termed as “parcels” in the following) with different levels of information content, which leads to having low-/high-dimensional spaces depending, e.g., on the results of a principal component analysis (PCA) applied to the raw data.

Finally, the third experiment focuses on the performance as a function of the length of the time series. In particular, this simulation study allows one to understand the potential difference between using $\textrm{MACB}_{X,X,Z}$ or relying on $\textrm{ACB}_{X,X,Z}$ in case of either short-time data analyses (such as those used in near real-time scenarios^33^).

In each of these three experiments, MACB is directly applied to two multivariate time series $\textrm{data}_X\in {\mathbb {R}}^{N_X\times T}$ and $\textrm{data}_Z\in {\mathbb {R}}^{N_Z\times T}$, while two ACB approaches (one with the same normalization as in the MACB formulation, i.e., as shown in Eq. (3), and one using the univariate normalization defined in Shahbazi et al.^25^) were separately applied to the two univariate time series $\textrm{data}_x\in {\mathbb {R}}^{T}$ and $\textrm{data}_z\in {\mathbb {R}}^{T}$ that come out from the application of PCAs to $\textrm{data}_X$ and $\textrm{data}_Z$. In order to assess the statistical significance of the difference between MACB performance and the performance of the two ACB-based approaches, we firstly obtained, for each synthetic experiment, three method-specific distributions (i.e., one distribution for MACB and two for ACBs) of the results in case of noise-only condition; we did not change the value of the other parameters. Second, we looked at these distributions and selected, for each method, the values associated with the 95-th percentile. Finally, we used these values as thresholds above which a coupling is considered to be significantly detected by the related method and relied on the fractions of total iterations above threshold of significance as performance indicator for each method.

In the first experiment, for 11 different levels of signal-to-noise ratio (SNR), i.e., from a noise-free to a noise-only situation, we generated 1000 3D time series $X\in {\mathbb {R}}^{3 \times T}$ of $T=$ 46,080 time bins (3 min length with the sampling frequency of 256 Hz) where each component was sampled from a i.i.d. process *N*(0, 10). Each 3D time series was then zero-phase fourth-order Butterworth filtered in a narrow (± 0.1 Hz) frequency band around 10 Hz, which in the neuroscientific field is known as the $\alpha$ peak, and quadratically coupled to itself in order to simulate a within-source cross-frequency interaction, i.e.,

$$\begin{aligned} {\tilde{X}}(t)=(1-w_{nl})\frac{X(t)}{\left\| X\right\| _F} +w_{nl}\frac{X^{\circ 2}}{\left\| X^{\circ 2}\right\| _F}, \end{aligned}$$

where $\circ$ denotes the Hadamard product. Similarly, ten additional 3D time series $N_i\in {\mathbb {R}}^{3 \times T}$ were generated relying on the above described process and simulating the noise sources. The only exception is that each noise source had a standard deviation of 0.1 (corresponding to a contribution of a 10% of noise for each simulated source). A linear matrix transformations *M* with standard Gaussian entries was applied to a delayed (with a lag $\tau =7$ bins, which approximately corresponds to 30 ms) copy of ${\tilde{X}}$ to define the time series *Z* associated with the 3D source coupled with the first one, i.e.,

4 $$\begin{aligned} Z(t)=M{\tilde{X}}(t+\tau ). \end{aligned}$$

The cortical locations of the two interacting regions, as well as the position of the ten noise sources, were chosen to be randomly located in the source space (a cortical layer of 8004 uniformly distributed points). The simulated time series were projected to the sensor space (composed of $C=256$ channels) to obtain the MEG recordings through the normalized lead fields *L* obtained with the single-shell approach^34^ for the source space and volume conductor model provided by the Human Connectome Project database^20,35^. In particular, the synthetic sensor time series $S\in {\mathbb {R}}^{C\times T}$ were defined as

$$\begin{aligned} S=(1-\gamma )\frac{L_{{\tilde{X}}}\tilde{X}+L_{Z}Z+\sum _{i=1}^{10}L_{N_i}N_i}{\left\| L_{{\tilde{X}}}\tilde{X}+L_{Z}Z+\sum _{i=1}^{10}L_{N_i}N_i\right\| _F} +\gamma \frac{\mu }{\left\| \mu \right\| _F} \end{aligned}$$

where $\mu \in {\mathbb {R}}^{C\times T}$ is a multivariate (noise) time series whose components are selected from a standard Normal Distribution, $L_{{\tilde{X}}}\in {\mathbb {R}}^{C \times 3}$ is the lead field of the location associated with the time series ${\tilde{X}}$ and $\gamma \in [0,1]$ denotes the weight of the sensor noise (i.e., from sensor noise-free to noise-only case). The reconstructed electrical source activities were then estimated at the true cortical sites (i.e., the known location of the interacting sources) relying on the inverse method with free source orientation termed exact low resolution brain electromagnetic tomography (eLoreta^31^). We relied on the eLoreta implementation provided by the FieldTrip MATLAB toolbox^36^. Therefore, by terming $W_X \in {\mathbb {R}}^{3 \times C}$ and $W_Z\in {\mathbb {R}}^{3 \times C}$ the rows (associated with the true position of the two interacting sources) of the matrix obtained by using the eLoreta inverse method, we had $\textrm{data}_X=W_XS$ and $\textrm{data}_Z=W_ZS$. As opposed to the first experiment, in the second one we directly investigated the effect of the dimensionality of the data spaces on MACB performance by varying the number of spatial components of the vector time series *X* and *Z* from $N_X=N_Z=1$ to $N_X=N_Z=10$ (thus, modeling cases ranging from univariate interacting sources to 10-dimensional coupled ones). The definition of *X*, ${\tilde{X}}$ and *Z* followed the same procedure used in the first experiment. To simulate correlated noise without using forward/inverse modelling, the vector noise time series $N_i$, whose simulation basically relied on the process described in the previous paragraph, were linear transformed into ${\tilde{\mu }}_{X}\in {\mathbb {R}}^{N_X \times T}$ and ${\tilde{\mu }}_{Z}\in {\mathbb {R}}^{N_Z \times T}$ using mixing matrices $M_{X}$ and $M_{Z}$ with suitable size and standard normal entries. $\textrm{MACB}_{X,X,Z}$ and the univariate methods (after using the dimensionality reduction approaches) $\textrm{ACB}_{X,X,Z}$ were then applied to

$$\begin{aligned} \textrm{data}_{X,Z}=(1-\gamma )\frac{{\tilde{X}},Z}{\left\| [{\tilde{X}}; Z] \right\| _F}+\gamma \frac{{\tilde{\mu }}_{X,Z} }{\left\| [{\tilde{\mu }}_{X}; {\tilde{\mu }}_{Z} ]\right\| _F}. \end{aligned}$$

Finally, when the effect of data length was at target, the number of spatial components of the vector time series was set to be equal to the one used in the first experiment, i.e., to 3. Nevertheless, the data length varied among the following values: 2 s, 5 s, 10 s, 30 s, 1 min, 3 min and 5 min.

## Results

**Figure 2:**
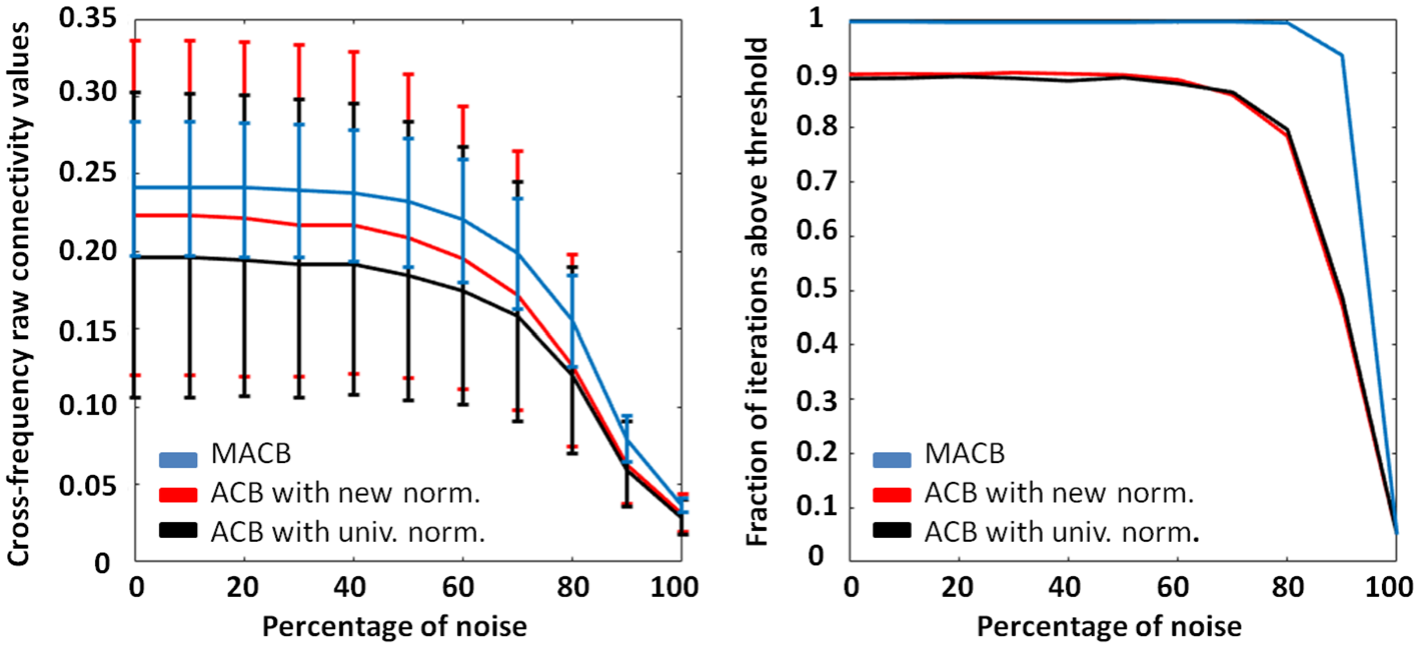
Left: the median and interquartile range of the raw connectivity values of the methods (blue line for MACB, and red and black lines for the two ACB approaches) for each simulated SNR condition (a percentage of noise equal to 0$\%$ indicates a noise-free situation while a $100\%$ denotes a noise-only case) for the first synthetic experiment. For each simulation iteration, the methods have been applied to two three-dimensional (source) time series obtained by relying on an electromagnetic forward and inverse (eLoreta) procedure. Right: the fractions of simulation iterations above thresholds of significance (e.g., 1 indicates that all the couplings are detected, while 0 denotes the lowest coupling detection rate, i.e., no detections).

The left panel of Fig. 2 shows the median (solid line) and the interquartile range (error bars) of the raw values obtained by the methods for each percentage of noise (0$\%$ denotes a noise-free case, $50\%$ a balance between noise and signal and $100\%$ a noise-only condition) within the first experiment (i.e., couplings between two synthetic 3D time series of 3-min length, with electromagnetic forward/inverse modelling). Although the method-specific raw values (that is the MACB and the ACB connectivity value) cannot be directly compared among them (e.g., a 0.5 obtained by MACB may not exactly correspond to a 0.5 of ACB, since the related statistical significance may not be the same), observing how they vary across and within SNRs may help in the interpretation of the difference among the performance. For instance, except for the noise-only case, it is evident that MACB (blue curve) is associated with the lowest variability (i.e., smallest error bars) for a fixed percentage of noise. This low variability allows the method to reach a higher coupling detection rate. This rate is shown in the right panel of Fig. 2 in terms of the fractions of simulation iterations above thresholds (e.g., 1 indicates that all the simulated couplings are detected, while 0 denotes no detections). Specifically, whereas the two (red/black) solid curves associated with ACBs almost coincide between them, the blue line rapidly tends to saturate to 1, thus indicating that MACB detects the presence of a true coupling even if the SNR is low. Hence, the statistical performance of MACB is better than the performance of the other two 1D methods. When a realistic inverse problem is not taken into account, i.e., when both the forward and the inverse matrices coincided with the identity matrix, similar differences among the methods can be obtained.

**Figure 3:**
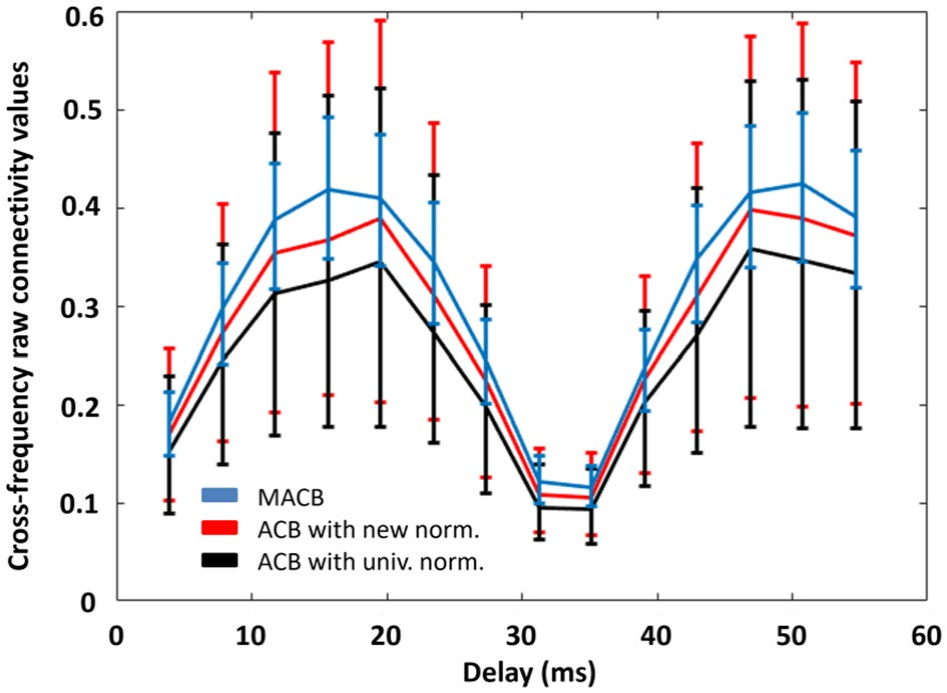
Cross-frequency connectivity values were obtained from MACB and its two corresponding one-dimensional methods. The results demonstrate fluctuations in values depending on the lag. MACB shows a smaller interquartile range compared to the 1D methods, which are adversely influenced by the multidimensional nature of the simulation. However, there are still lag values and, consequently, phase difference values, preventing any of the analyzed methods from achieving values significantly different from those obtained for a 0-lag interaction (which is intrinsically indistinguishable from an artefactual coupling).

In order to extrapolate information about the influence of the lags on the connectivity values, we also analyzed the methods output as functions of the time delay in the interaction. In particular, we varied the lag from 4 to 55 ms in the Eq. (4). The results (Fig. 3) clearly show that the MACB and ACBs values oscillate (with a period depending on the interaction model, e.g., frequency bands at play) as a function of the delay. Two key points emerge. Firstly, MACB exhibits a smaller interquartile range than the 1D methods, which are negatively affected by the multidimensional nature of the coupling. Secondly, there are lag values (e.g., around 35 ms in this interaction model) and, consequently, related phase difference values, such that none of the analyzed methods can achieve values that are dissimilar from those obtained for a 0-lag interaction (indistinguishable from an artifact coupling). However, this is a drawback generated by (mathematical) construction and unavoidable given the intention for the methods (both MACB and ACB) to be robust to artificial instantaneous coupling.

**Figure 4:**
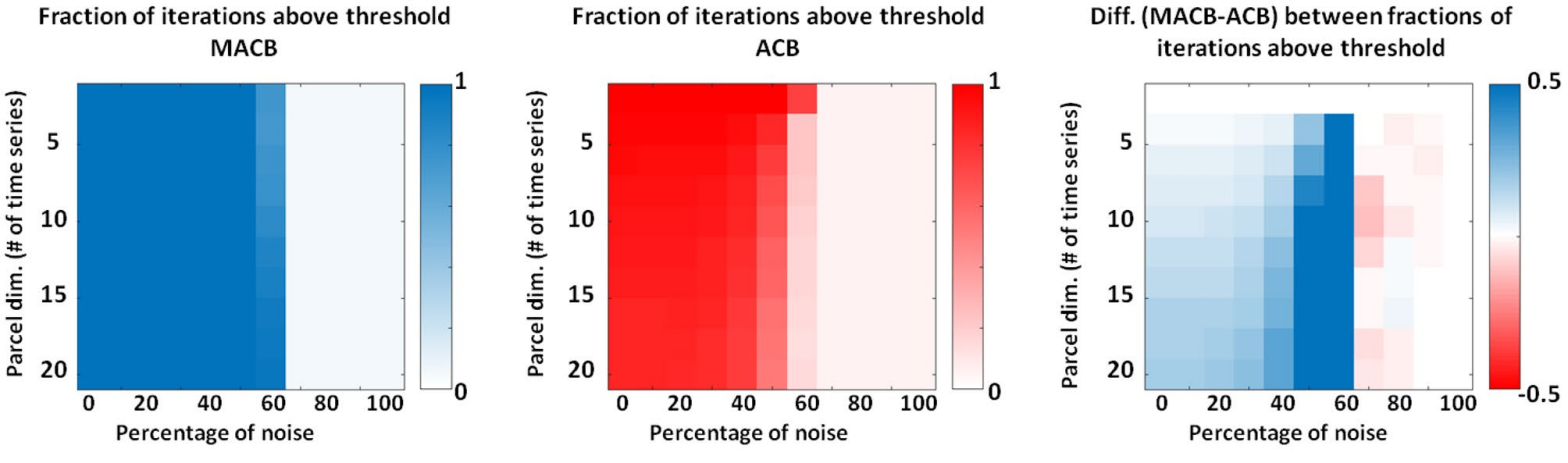
Left and middle: the fractions of simulation iterations that exceeded the method-specific (MACB in blue and ACB with the new normalization in red) threshold of significance, i.e., the coupling detection rate for the methods for each of SNR situation and each data space dimension. A data space dimension of 20 denotes the case in which each of the two (coupled) vector time series has 10 components. Right: the difference between the detection rate of the two methods.

For the second synthetic experiment, Fig. 4 shows the colour-coded fractions of simulation iterations above thresholds reached by MACB (left panel) and by an ACB approach (middle panel), and the difference between the former and the latter (right panel). Viewed through a neuroscientific lens, the second experiment aimed to simulate the occurrence of cross-frequency coupling among brain regions with various dimensionalities of the corresponding data spaces. These different dimensionalities may result, for instance, from the principal component analyses (PCA) applied to the data. Similarly to what is shown in Fig. 2, a constantly high coupling detection rate followed by a rapid decrease of the fractions of iterations above threshold is evident for both of the two methods considered. In particular, almost independently on the data space dimension, MACB performance starts to decrease when SNR < 1 (i.e., when the percentage of noise in the model exceeds the weight of the signal, i.e., percentage of noise higher than $50\%$); the ACB curve shows a higher dependency on the dimensionality, which clearly reflects the fact that the information loss (using only the first PCA component) increases with the increase of the data space dimension, thus leading to lower performance for higher dimension. The difference (MACB–ACB) in the behavior is evident in the right panel: starting from a percentage of noise equal to $0\%$, and below a percentage of $70\%$, the value of this difference increases with the decrease of the SNR and with the increase of the data space dimensionality.

**Figure 5:**
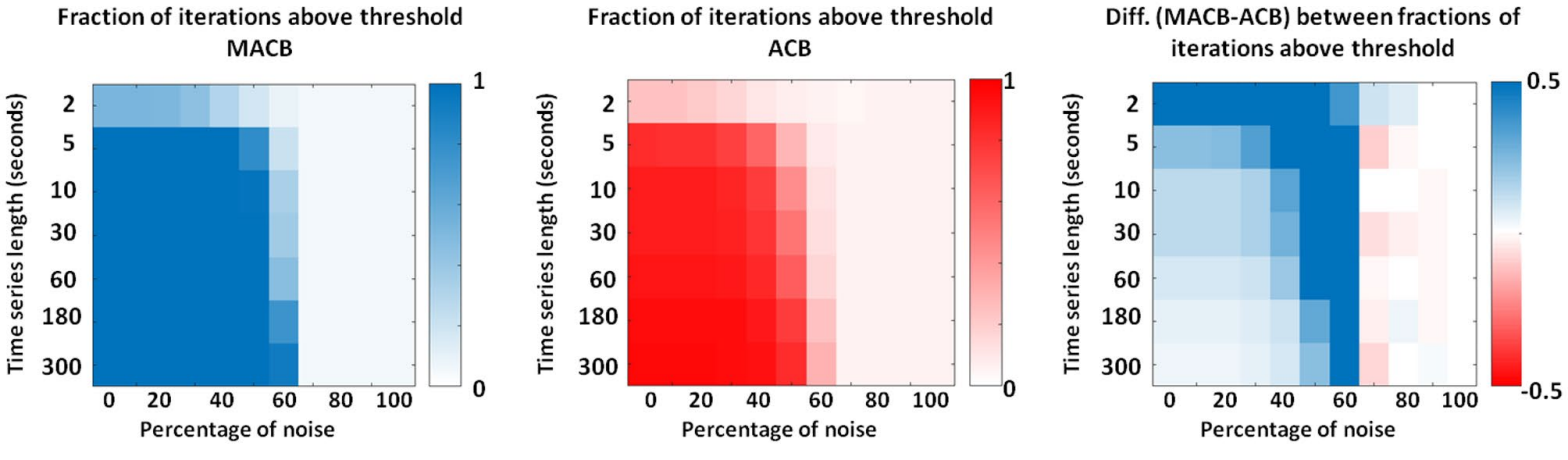
Left and middle: the fractions of simulation iterations that exceeded the method-specific (MACB in blue and ACB with the new normalization in red) threshold of significance, i.e., the coupling detection rate for the methods for each of SNR situation and each data length. A time series length of 3 s denotes the case in which each of the two (coupled) three-dimensional time series has a number of data bins equal to $3 \cdot 254$, where 254 Hz is the sampling frequency. Right: the difference between the detection rate of the two methods.

For the third synthetic experiment, Fig. 5 shows the method-specific fractions of simulation iterations above threshold of significance. Considering a neuroscientific standpoint, this experiment provided an opportunity to assess the effectiveness of employing methods for conducting a dynamic (i.e., over time) cross-frequency connectivity analysis on short-length vector data. In general, similarly to Fig. 4, the fractions of iterations above threshold of significance is high for SNR > 1 and starts to rapidly decrease when the noise percentage exceeds 50$\%$ (left and middle panels). As opposed to the results related to the previous experiment, the coupling detection rate is strongly dependent on the other analysed variable, i.e., on the time series length. Indeed, while for a data length larger than (or equal to) 10 s, MACB performance is higher than that of ACB only for $\textrm{SNR}\approx 1$; for shorter data, the difference between the methods is also evident at $\textrm{SNR}=\infty$ (right panel of Fig. 5).

## Discussion

We introduced a generalisation to the multivariate case of a statistical method based on bispectral analysis, termed antisymmetric cross-bicoherence (ACB^24,32^). The novel index is called multi-dimensional antisymmetric cross-bicoherence (MACB). MACB is a blockwise approach to analyse pairwise coupling consisting of non-linear (quadratic) lagged phase interactions between vector time series in the frequency domain. Cross-frequency interactions are well-known in the functional neuroimaging field, where they can be empirically found in electrophysiological data^14^. Specifically, it has been hypothesised that this coupling modality is a mechanism implemented by the brain for integrating information across different spatial and temporal scales^16,17^. In this framework, the exploitation of a multi-dimensional method may improve the detections of functional connectivities through an avoidance of a dimensionality reduction that would lead to an information loss^3,37–39^.

It is important to notice that we used the term “multi-dimensional” for MACB to prevent potential confusion with some of the meanings related to the term “multivariate”. Indeed, in the literature, the adjective “multivariate” also carries meanings that are inconsistent with what we were referring to. For instance, it may also denote the estimation of (one-dimensional) dependencies between all pairs of nodes within a network. This can involve techniques such as multivariate autoregressive modeling^40^ or, from a certain perspective, pairwise interacting source analyses^24^; accordingly, to steer clear of ambiguity, the term “multi-dimensional” may be used to refer to the estimation of a single connection between nodes composed of multiple time series^1,3^. Clearly, the challenge of disentangling all cross-frequency dependencies between subnetworks cannot be addressed through MACB, since it has been specifically formulated to either detect pairwise (first source *X* at $f_1$ and $f_2$, second source *Z* at frequency $f_1+f_2$) or tripletwise (first source *X* at frequency $f_1$, second source *Y* at $f_2$ and third source *Z* at frequency $f_1+f_2$) dependencies. In future work, it could be interesting to develop a method that preserves the fundamental properties of MACB (e.g., its invariance under rotation of the data) and that can detect multivariate dependencies among all the pairs/triplets of blocks of time series, thereby bridging two meanings of “multivariate”.

The value of MACB lies between 0 and 1, and it is associated with a bias that for Gaussian data decreases approximately with the square root of the number of trials in which the data are divided (see Fig 1). Furthermore, the introduced method is invariant under orthogonal trasformations of the data. The latter property makes it independent on, e.g., the choice of the physical coordinate system used in the electromagnetic inverse process. Indeed, prior to applying MACB to electro/magnetoencephalographic (EEG/MEG) data, the scalp level signals have to be projected into a source space/grid that models the cortex in order to estimate the neural generators of the measured data, but this procedure requires choosing an arbitrary coordinate system^10,31^; therefore, a method that is independent of this choice is fundamental. In addition, the accuracy of coregistration between the measurement array and the subject MRI influences the reliability of such a reference system across subjects and, in turn, functional connectivity estimates^41^.

In extensive and biologically realistic simulations, we experimentally showed that MACB reached significantly better results than its one-dimensional version. In the first synthetic experiment, the emphasis was on evaluating the divergence between the two methods in relation to the noise level within a model designed to simulate a realistic neuroscientific scenario (see Figs. 2, 3). When an inverse problem was not considered, the same differences among the methods can be observed. This suggests that the selection of the inverse method does not significantly affect MACB any more than it affects its 1D version.

It is worth to notice that, in the performed analyses, the value chosen as a threshold above which an iteration was considered as significant corresponded to the single tail 95th percentile, meaning that $95\%$ of the simulation iterations in the case of only noise were below this value. This selection was made to limit the number of false positives to a conventional percentage, i.e., $5\%$, and to assess the differences in terms of true positive detections. Nevertheless, by choosing other values, e.g., 99th percentile, the obtained results essentially coincided with those related to a higher threshold (except for a general reduction in performance for low SNR levels due to the reduction in the tolerated false positives). This also demonstrated that, for high SNR situations, the percentage of false positives necessary to achieve perfect performance in terms of true positive detections is less than $1\%$ for MACB, while it is over $50\%$ for ACB approaches. Regarding the statistical methodology employed, it is crucial to highlight that its functioning is closely tied to the fact that these were synthetic data, and the ground truth was perfectly known. In real data, using a similar statistic or analyzing receiver operating characteristics is impossible due to the lack of information regarding true/false positives. In the case of real data, it is advisable, for example, to use a contrast between different experimental conditions across subjects corrected via a cluster-based permutation approach^42^.

From a neuroscientific point of view, the second experiment modelled the presence of cross-frequency coupled brain regions for various dimensionality of the associated data spaces (which may depend, e.g., on the results of principal component analyses—PCA applied to the data). In particular, the higher the dimension of the single data space the higher the difference between MACB and ACB performance (see Fig. 4). The increasing difference in the performance should be taken into account when the regions of the cortex are suitably clustered into different parcels based on either functional or structural information; indeed, some parcels may be dimensionally large compared to the others, and they may be associated with a high number of time series even if a PCA is applied. This would lead to a large performance difference between MACB and ACB.

Instead, the third synthetic experiment allowed us to analyse the efficiency of using the methods for performing a dynamic (i.e., over time) cross-frequency connectivity analysis between short-length vector data. Even if more complex in nature than standard functional connectivity investigation, it is worthwhile to consider such a scenario, as analyzing the fluctuations in connectivity patterns over time in real data may reveal additional insights into the brain functional mechanism that cannot be understood otherwise^33,43^. Specifically, we found that the shorter the data length the higher the difference between MACB and ACB performance (see Fig. 5). These findings allow one to understand that the application of a dimensionality reduction approach for short-length data induces a significant information loss that negatively impacts the performance of ACB (compared to MACB) even for a high SNR.

In future works, it will be of interest to exploit the MACB for detecting the presence of quadratic cross-frequency coupling in real data, e.g., those taking from the Human Connectome Project^35^, in order to overcome the issues of the one-dimensional (e.g., potential suboptimality in terms of information loss) methods.

## Data Availability

The datasets used and/or analysed during the current study available from the corresponding author on reasonable request.
